# Picture perfect constrictive pericarditis: a case report

**DOI:** 10.1093/ehjcr/ytag181

**Published:** 2026-03-10

**Authors:** Mohamad Bahrou, Soomal Rafique, Karthik Missula, Mohsin Salih

**Affiliations:** Department of Internal Medicine, Southern Illinois University School of Medicine, 751 N. Rutledge, Springfield IL 62702, USA; Department of Internal Medicine, Southern Illinois University School of Medicine, 751 N. Rutledge, Springfield IL 62702, USA; Division of Cardiology, Department of Internal Medicine, Southern Illinois University School of Medicine, 751 N. Rutledge, Springfield IL 62702, USA; Division of Cardiology, Department of Internal Medicine, Southern Illinois University School of Medicine, 751 N. Rutledge, Springfield IL 62702, USA

**Keywords:** Case report, Computed tomography, Echocardiography, Imaging, Magnetic resonance imaging, Pericarditis, Constrictive

## Abstract

**Background:**

Constrictive pericarditis (CP) is a rare complication of acute pericarditis, caused by pericardial fibrosis and calcification that impair diastolic filling. It leads to right-sided heart failure and requires multimodal imaging for diagnosis, including echocardiography, cardiac magnetic resonance imaging, and right heart catheterization.

**Case summary:**

A 75-year-old male with a recent history of pneumonia and chest pain presented with dyspnoea and oedema. Echocardiography showed a septal bounce; CT revealed pericardial calcification; and right heart catheterization demonstrated equalized diastolic pressures with a square root sign. Magnetic resonance imaging confirmed CP. The patient was managed medically and referred for pericardiectomy.

**Discussion:**

This case illustrates classic CP with hallmark clinical, imaging, and haemodynamic findings. It emphasizes the diagnostic value of multimodal evaluation and serves as a comprehensive example of this uncommon but critical condition.

Learning points

- Multimodal diagnosis: Constrictive pericarditis (CP) diagnosis requires echocardiography, cardiac magnetic resonance imaging, and right heart catheterization to identify key features like septal bounce and equalized diastolic pressures.
- Clinical presentation: The most common symptom of CP is dyspnoea, with additional symptoms including hepatomegaly, jugular venous distension, ascites, peripheral oedema, pleural effusions, and Kussmaul’s sign.

## Introduction

Constrictive pericarditis (CP), a rare complication of acute pericarditis, is associated with high morbidity and mortality. It can cause diastolic heart failure^1^—use of anti-inflammatory therapy is effective for transient CP and pericardiectomy for chronic cases. Key diagnostic features include careful assessment of the jugular venous pulse, pericardial knock, and clinical presentation. The pathophysiology of CP involves dissociating intrathoracic and intracardiac pressures and interventricular dependence.^2^ We present a case with classic clinical presentation, typical radiologic, echocardiographic and cardiac catheterization findings of CP, and refreshing pathophysiological basics.

## Case presentation

### Initial presentation

A 75-year-old man with a history of hypertension, hyperlipidaemia, class II obesity, and a 40-pack-year smoking history presented to the cardiology clinic with progressive shortness of breath on minimal exertion and marked bilateral lower extremity oedema. Four months earlier, he experienced sharp, pleuritic chest pain that improved with shallow breathing and was treated as pneumonia. Although the chest pain resolved, his dyspnoea persisted and gradually worsened. He was subsequently referred to the hospital for further evaluation and management of suspected pericardial disease.

### Vitals and examination

Upon admission, his physical examination was notable only for mild lower extremity oedema. He was afebrile, with a blood pressure of 148/85 mmHg, a normal heart rate of 62 b.p.m., a respiratory rate of 18 breaths per minute, and oxygen saturation within the normal range. An EKG showed a first-degree AV block with a right bundle branch block.

### Investigations and clinical course

**Figure 1:**
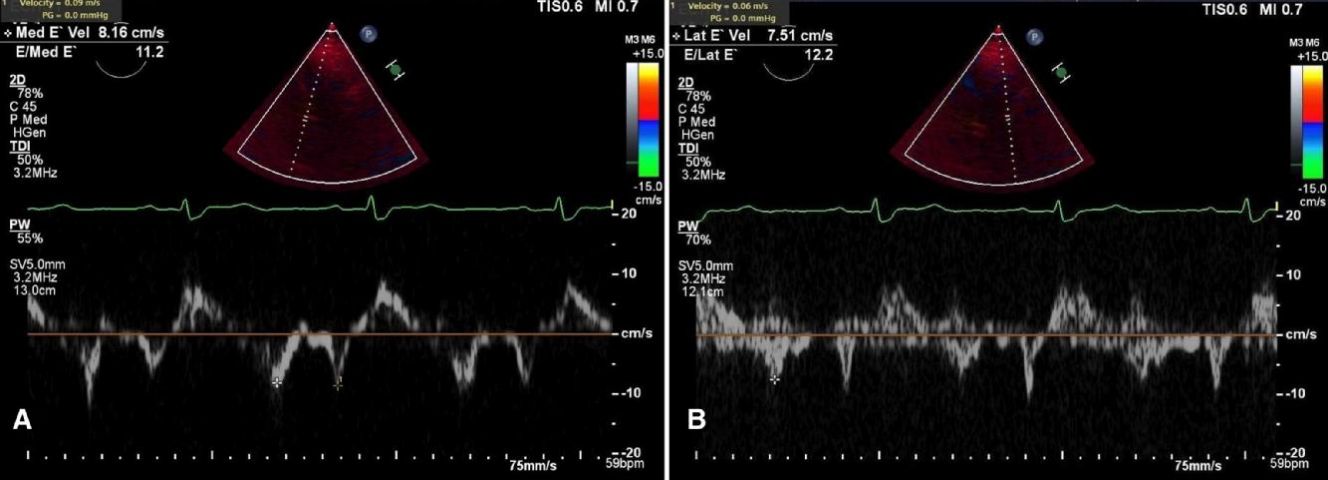
Transthoracic echocardiogram. (*A*) Medial mitral annular velocity and (*B*) lateral mitral annular velocity demonstrating *annulus reversus*.

**Figure 2:**
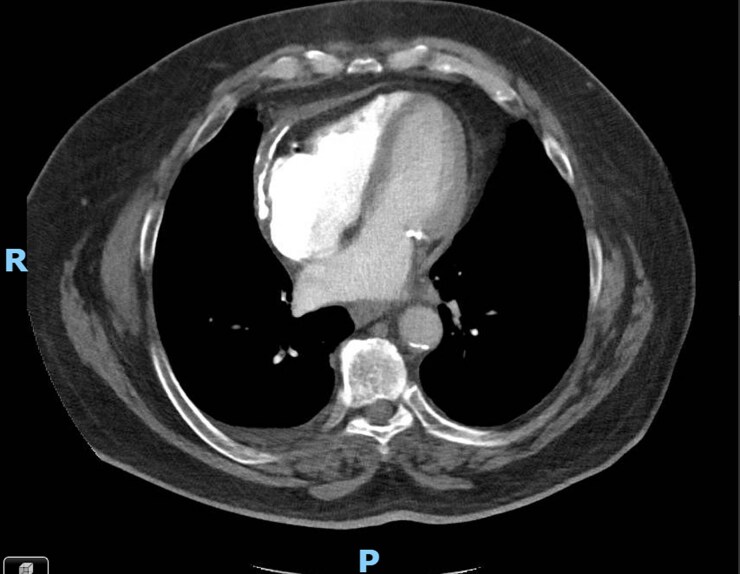
Computed tomography angiography of the chest showing pericardial calcifications overlying the right ventricle.

**Figure 3:**
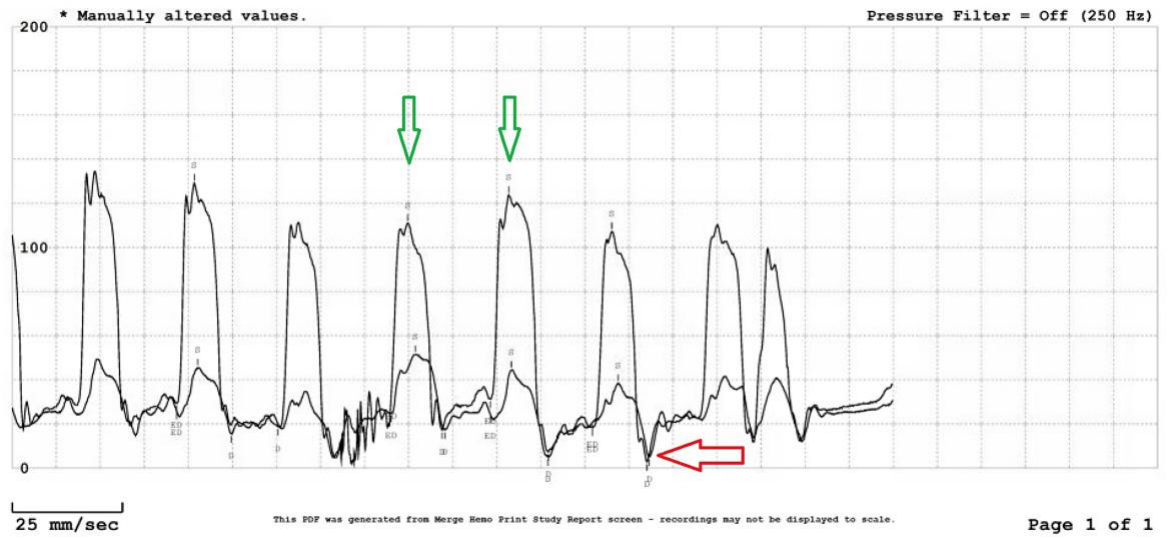
Right heart catheterization demonstrating equalization of left and right ventricular diastolic pressures, left ventricular and right ventricular (green arrow) discordance, and the characteristic ‘square root’ sign of right ventricular diastolic pressure (red arrow).

**Figure 4:**
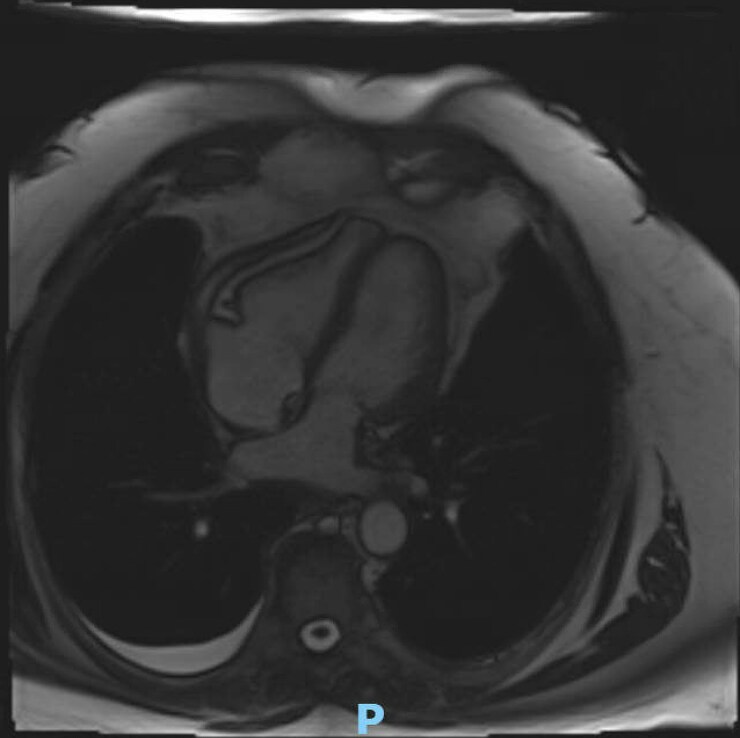
Cardiac magnetic resonance imaging showing pericardial thickening with septal flattening.

Initial laboratory investigations were largely unremarkable, except for an elevated C-reactive protein (7.7, N: <1 mg/dL). A transthoracic echocardiogram (TTE) showed a normal left ventricular ejection fraction, a septal bounce suggestive of CP, an E/A ratio of 2.39, medial and lateral mitral annular velocities of 9 cm/s and 6 cm/s, an *E*/*e*′ ratio of 12.2 (see Supplementary material online, *Video S1*; *Figure 1*). Computed tomography angiography (CTA) demonstrated severe pericardial calcifications overlying the RV (*Figure 2*). Right heart catheterization revealed discordance between the left ventricular (LV) and right ventricular (RV) pressures, equalization of LV and RV diastolic pressures, and the square root sign of RV diastolic pressure (*Figure 3*). Cardiac magnetic resonance imaging (CMR) showed pericardial thickening with septal flattening and a septal bounce, suggesting CP and reduced RV function (seeSupplementary material online, *Videos S2* and *S3*; *Figure 4*). *Table 1* shows relevant cardiac imaging and cardiac catheterization findings.

**Table 1:** Relevant cardiac imaging and cardiac catheterization findings

Cardiac imaging	Finding
CT chest	Pericardial calcification and increased thickness
Transthoracic echocardiogram	Ventricular septal bounce Medial mitral annulus *e*′ velocity ≥ 9 cm/s Restrictive mitral inflow velocity (E/A ratio > 0.8)
Cardiac MRI	Ventricular septal bounce Pericardial thickening Myo-pericardial tethering Conical ventricular formation Diastolic resistance
Cardiac catheterization	Equalization of the diastolic pressure across all four chambers ‘Dip-and-plateau’ pattern Right atrial pressure: 22 mmHg Wedge pressure was 24 mmHg

The patient was managed with colchicine, aspirin, and IV diuresis. Cardiothoracic surgery recommended pericardiectomy. Due to the high surgical risk and the potential need for right ventricular support, the patient was transferred to a tertiary care centre for surgical management. At his most recent follow-up, 11 months after undergoing a successful pericardiectomy, he remained asymptomatic with no post-operative complications.

## Discussion

Constrictive pericarditis is a disease characterized by a rigid and stiff pericardium resulting from dense fibrosis and calcifications due to long-standing pericarditis. This stiff pericardium impairs heart diastolic function, leading to right heart failure without pulmonary oedema. Constrictive pericarditis following acute pericarditis has been reported in 1.8% of cases.^1^ Constrictive pericarditis can arise from various aetiologies, including idiopathic or viral (42%–49%), post-cardiotomy (11%–37%), post-radiation (9%–31%), connective tissue diseases (3%–7%), post-infectious causes including tuberculosis (3%–6%), and other uncommon causes (<10%) such as neoplasia, thoracic trauma, uraemic pericarditis, and sarcoidosis. It typically takes between 6 and 12 months from the onset of symptoms to make the diagnosis of CP. The most common symptom is dyspnoea, but other symptoms are also prevalent, including hepatomegaly (23.4%–100%), jugular venous distension (52%–65%), ascites (8.9%–90%), and peripheral oedema (8.9%–84%). Pleural effusions (35%–79.3%) and Kussmaul’s sign have been reported in 63.3% of cases.^3^

According to the European Society of Cardiology guidelines, it is best to use a multi-modality approach, including TTE, CMR, and cardiac catheterization to diagnose and differentiate CP from other conditions.^4^ Transthoracic echocardiogram is considered the initial approach due to its easy accessibility, where a set of features may aid in the diagnosis of CP, including ventricular septal bounce (seen in 75% of patients with CP), medial mitral annulus *e*′ velocity ≥ 9 cm/s, and hepatic vein expiratory diastolic reversal ratio ≥ 0.79, in addition to restrictive mitral inflow velocity (E/A ratio > 0.8) and a plethoric inferior vena cava (dilated or minimal collapse during inspiration). These findings, in combination, accounted for 87% sensitivity and 91% specificity in one study. Other findings noted on TTE were tethering of the right ventricular free wall to the liver and distorted contours of the left and right ventricles due to calcified pericardium.^5,6^ There have been no established guidelines to diagnose CP on CMR, but a set of features has been observed, including pericardial thickening, myo-pericardial tethering, conical ventricular formation, diastolic resistance, ventricular septal bounce, and dilated inferior vena cava. Additionally, pericardial oedema and calcification reflect the persistent inflammation process.^7^

Cardiac catheterization remains the gold standard diagnostic modality for diagnosing CP by evaluating intracavitary pressure changes and identifying certain patterns. Equalization of the diastolic pressure across all four chambers of the heart is one such finding, which reflects a stiff, calcified pericardium that limits diastolic volume in all chambers. However, this finding is typically seen only in diffuse CP. Another recognized pattern is the ‘dip-and-plateau’, where a plateau follows rapid filling of the ventricles. Prominent ‘x’ and ‘y’ descent pattern in the jugular venous pulse and right atrium (RA) is well described in the literature, which happens because of rapid RA emptying during the early phase of diastole.^8^

Diagnosing CP is challenging and relies on clinical symptoms of right-sided heart failure, along with a multimodal approach that includes echocardiography, CMR, and cardiac catheterization, which is considered the gold standard diagnostic modality. Our patient presents as a classic case of CP, exhibiting typical signs and symptoms. Radiological findings also align with those commonly described in the literature. Given the rarity of CP, this case serves as an excellent example of the condition, showcasing all the key features documented in medical studies. It provides a comprehensive overview of CP and a valuable refresher on its pathophysiologic fundamentals.

## Supplementary Material

ytag181_Supplementary_Data

## Data Availability

All data supporting the findings of this case report are included within the article. No additional data are available.

## Lead author biography

Dr Mohamad Bahrou is an internal medicine resident in the USA with a strong interest in cardiology. He is involved in research focused on cardiovascular imaging and complex cardiac conditions. He aims to pursue a cardiology fellowship and integrate research into clinical care. Outside of medicine, he enjoys teaching and academic writing.

## Notes

**Consent:** Informed consent was obtained from the patient for publication of this case report and any accompanying images. This publication complies with the COPE (Committee on Publication Ethics) guidelines.
